# Association of Germline Single Nucleotide Polymorphisms in Steroid Hormone Metabolism Pathway With Androgen Deprivation Therapy Prognosis of Prostate Cancer in Chinese Population

**DOI:** 10.1002/cam4.71351

**Published:** 2025-11-02

**Authors:** Ruofan Shi, Xiaohao Ruan, Qijun Du, Tsun Tsun Stacia Chun, Da Huang, Kuen Chan, Yuguang Philip Wu, Tsz Yeung Kam, Danfeng Xu, Rong Na

**Affiliations:** ^1^ Department of Urology Ruijin Hospital, Shanghai Jiao Tong University School of Medicine Shanghai China; ^2^ Division of Urology, Department of Surgery, School of Clinical Medicine, LKS Faculty of Medicine The University of Hong Kong Hong Kong China; ^3^ Department of Clinical Oncology Pamela Youde Nethersole Eastern Hospital Hong Kong China

**Keywords:** androgen deprivation therapy (ADT), prostate cancer (PCa), single nucleotide polymorphism (SNP), steroid hormone metabolism

## Abstract

**Background:**

Single nucleotide polymorphisms (SNPs) located in the genes participating in the steroid hormone metabolism pathway may influence the outcomes of androgen deprivation therapy (ADT) in prostate cancer (PCa) patients, but findings on the Chinese population remain limited.

**Methods:**

A multicentric cohort of 301 Chinese PCa patients receiving first‐line ADT was enrolled. Germline SNPs located in 62 steroid hormone metabolism‐related genes were analyzed for associations with time to ADT failure using multivariate Cox regression. Important expression quantitative trait loci (eQTLs) were discovered.

**Results:**

Four SNPs were significantly associated with time to ADT failure: rs36119043 in *AKR1D1* (hazard ratio, HR = 2.02, 95% confidence interval, 95% CI: 1.44–2.85, *p* = 5.72 × 10^−5^), rs151155810 in *HSD17B12* (HR = 7.87, 95% CI: 2.78–22.30, *p* = 1.05 × 10^−4^), rs71179009 in *SULT2B1* (HR = 2.16, 95% CI: 1.44–3.22, *p* = 1.85 × 10^−4^), rs28609134 in *SRD5A3* (HR = 2.50, 95% CI: 1.51–4.15, *p* = 3.79 × 10^−4^). Potential causal eQTLs in the LD regions of these SNPs were identified, with significant impacts on *AKR1D1*, *SULT2B1*, and *SRD5A3* expression in diverse tissues. A cumulative risk allele effect was observed: HR = 2.74 (95% CI: 1.86–4.03) under the dominant model and HR = 2.04 (95% CI: 1.63–2.55) under the additive model, with a median survival of 176 months (95% CI: N/A) in noncarrier patients vs. 92 months (95% CI: 65–N/A) in one risk locus‐carriers and 55 months (95% CI: 26–N/A) in two risk loci‐carriers.

**Conclusions:**

SNPs in the steroid hormone metabolism pathway can predict time to ADT failure in Chinese PCa patients, supporting their potential role for drug response and pharmacogenomic stratification.

## 1 Introduction

Prostate cancer (PCa) is the second most commonly diagnosed malignant neoplasm and the fifth leading cause of cancer‐specific mortality among men worldwide [1]. It presents significant global challenges not only due to its rising incidence but also because of widening health disparities between countries and the increasing pressure on healthcare systems to ensure equitable access to screening and treatment [2]. In China, the incidence of PCa has also been steadily rising in recent years [3]. Androgen deprivation therapy (ADT) is the first‐line treatment for hormone‐sensitive PCa (HSPC). Despite initial responses, an enormous proportion of patients will finally progress to ADT failure, which represents the development of castration‐resistant PCa (CRPC) [4], which is a lethal disease stage with limited effective therapeutic options. The mechanisms underlying ADT resistance have been found to be multifactorial, involving excessive production of androgen, androgen receptor‐related factors [4], etc.

Given the central role of androgen in both PCa treatment and progression, genetic variations in key enzymes involved in androgen biosynthesis and metabolism may influence individual responses to ADT. In the past decades, several single nucleotide polymorphisms (SNPs) located in genes such as *CYP19A1*, *HSD3B1*, *HSD17B4*, and *SLCO1B3* have been implicated in ADT failure in Caucasian populations [5, 6]. In East Asian populations, including Japanese and Chinese cohorts, SNPs located in genes such as *AKR1C3*, *CYP17A1*, *HSD3B1*, and *SRD5A2* have also been reported to be associated with the efficacy of ADT [7, 8, 9]. However, evidence derived specifically from the Chinese population remains scarce. Moreover, most existing studies have examined isolated variants rather than taking a comprehensive perspective on the androgen metabolism pathway. The presence of more Chinesespecific variants and their effect sizes has not been systematically characterized. This highlights the need for more integrative genetic studies within Chinese cohorts to better understand the role of germline variation in modulating treatment response to ADT.

To address this gap, we conducted a multicentric observational study to investigate the association between germline SNPs located in steroid hormone metabolism‐related genes (*n* = 62) and time to ADT failure among Chinese PCa patients. We aimed to identify predictive SNPs that may contribute to interindividual differences in treatment response, thereby providing insights into the development of personalized therapeutic strategies.

## 2 Subjects and Methods

### 2.1 Study Population

This multicentric observational study comprises two sub‐cohorts: (1) a sub‐cohort derived from a previously described biopsy cohort [10], comprised of patients recruited from Shanghai Ruijin Hospital; (2) a consecutively enrolled cohort of PCa patients from Hong Kong, recruited at two tertiary medical centers: Queen Mary Hospital and Pamela Youde Nethersole Eastern Hospital. The patient recruitment process is illustrated in Figure S1. This study was approved by the ethics committees of all participating medical centers.

Inclusion criteria were as follows: (1) diagnosis of PCa in or before February 2024, confirmed either by prostate biopsy or by radiological methods; (2) receipt of first‐line ADT for HSPC, including gonadotropin‐releasing hormone (GnRH) analogues (e.g., leuprorelin, goserelin, triptorelin) or GnRH antagonists (e.g., degarelix); (3) provision of blood or buccal mucosa samples for germline SNP genotyping. Exclusion criteria included: (1) receipt of ADT as part of the adjuvant therapy following radical prostatectomy (RP) or as part of the doublet or triplet therapy (e.g., concurrent radiotherapy and/or chemotherapy with ADT); (2) unavailability of the follow‐up data; (3) diagnosis of other malignancies with concurrent active treatment. A total of 301 subjects met the above criteria and were finally included in the subsequent analyses. All participants provided written informed consent prior to enrollment.

### 2.2 Clinical Data Collection and Study Outcomes

Baseline clinical data collected included age at enrollment, baseline serum total prostate‐specific antigen (PSA) level, and Gleason grade group based on cancer pathology. Follow‐up at the Shanghai study center was conducted through scheduled outpatient clinic visits, supplemented by regular telephone interviews. In Hong Kong, the follow‐up relied on longitudinal electronic health records, including medical history, laboratory tests, and imaging findings.

The outcome of this study was the time to ADT failure, which was determined based on two criteria: (1) a sustained serum testosterone level < 50 ng/dL following initiation of ADT; (2) documented biochemical or clinical progression. Biochemical progression was defined as a serum total PSA level > 2 ng/mL, confirmed by at least two sequential rises of more than 50%, spaced a minimum of 1 week apart. Clinical progression was identified through imaging modalities such as computed tomography or bone scintigraphy, either showing at least two new metastatic foci or a ≥ 50% increase in the maximum diameter of existing lesions.

### 2.3 SNP Genotyping and Selection

The methods for SNP array genotyping, quality control, and imputation have been described elsewhere [10]. Variants with a minor allele frequency (MAF) < 1% or Hardy–Weinberg equilibrium *p* < 1 × 10^−6^ were excluded during quality control. SNP clumping was performed to account for linkage disequilibrium (LD), using a 1000 kb window and excluding SNPs in LD with the index SNP if the pairwise correlation coefficient r^2^ ≥ 0.2 or Lewontin's D' ≥ 0.8. All SNP quality control and LD clumping procedures were conducted using PLINK (version v1.90b7.2) [11] and the R package “LDlinkR” [12]. The data used for the expression quantitative trait loci (eQTLs) analyses were obtained from the Genotype‐Tissue Expression (GTEx) Portal (https://gtexportal.org, Release V10).

## 3 Statistical Analysis

Descriptive statistics were used to summarize baseline clinical characteristics. Continuous variables were summarized as medians with interquartile ranges and compared across sub‐cohorts using Student's *t*‐test for normally distributed variables and the Kruskal–Wallis test for non‐normally distributed variables. Categorical variables were summarized as frequencies and proportions and compared using Pearson's chi‐square test or Fisher's exact test, depending on cell counts. Ordinal variables such as the Gleason grade group were analyzed across sub‐cohorts using the Jonckheere–Terpstra trend test.

Cox proportional hazards regression models were used to evaluate the association between SNPs and ADT failure status. Cox models and Kaplan–Meier survival curves were implemented using R version 4.3.1 [13]. Differences in survival across genotype groups were assessed using the log‐rank test.

A two‐sided *p* value < 0.05 was considered statistically significant. A Bonferroni correction for the multiple comparisons was applied to control the inflation of the type I error in the genetic association analysis. As a result, the statistically significant level of the genetic association analysis was < 8.06 × 10^−4^ (0.05/62 significant SNPs after clumping).

## 4 Results

The baseline clinical characteristics of the 301 study participants are summarized in Table 1. The median follow‐up time was 28 months. No significant differences were observed among the sub‐cohorts in terms of the distribution of Gleason grade group. A total of 82 (27.2%) study subjects experienced ADT failure. Based on the KEGG steroid hormone biosynthesis pathway [14] and a review of the previous literature, 62 related genes were identified to be potentially related to ADT response (Table S1). From these genes, a total of 12,691 SNPs located within ±100 kb of their genomic regions were selected for analysis. After quality control, 7478 SNPs were retained for subsequent analysis.

**TABLE 1:** Baseline clinical characteristics of the study subjects.

Clinical characteristics^a^	Combined cohort (*n* = 301)	PYNEH^b^ (*n* = 25)	QMH^c^ (*n* = 62)	RJH^d^ (*n* = 214)	*p* ^e^
Median follow‐up (months), median (IQR)	28.0 (15.0–47.0)	25.0 (13.0–77.0)	17.0 (9.0–52.3)	29.0 (19.0–45.8)	**0.032**
Age at enrolment (years), median (IQR)	75.0 (68.0–81.0)	73.0 (64.0–78.0)	77.0 (70.0–81.0)	74.0 (68.0–81.0)	0.119
Serum total PSA before ADT (ng/mL), median (IQR)	24.3 (10.5–73.9)	71.0 (17.7–712.8)	52.0 (12.0–197.3)	20.0 (10.3–43.6)	**0.001**
Gleason grade group, *n* (%)					
1	25 (8.3)	5 (20.0)	5 (8.1)	15 (7.0)	*p* for trend = 0.159
2	48 (15.9)	2 (8.0)	5 (8.1)	41 (19.1)	
3	62 (20.6)	3 (12.0)	10 (16.1)	49 (22.9)	
4	67 (22.3)	2 (8.0)	10 (16.1)	55 (25.7)	
5	77 (25.6)	5 (20.0)	22 (35.5)	50 (23.4)	
N/A	22 (7.3)	8 (32.0)	10 (16.1)	4 (1.9)	
# of ADT failure, *n* (%)	82 (27.2)	14 (56.0)	17 (27.4)	51 (23.8)	**0.003**
# of radical prostatectomy, *n* (%)	100 (33.2)	3 (12.0)	9 (14.5)	88 (41.1)	**< 0.001**
# of radiotherapy, *n* (%)	32 (10.6)	10 (40.0)	8 (12.9)	14 (6.5)	**< 0.001**
# of chemotherapy, *n* (%)	7 (2.3)	1 (4.0)	2 (3.2)	4 (1.9)	0.517
# of distant metastasis, *n* (%)	105 (34.9)	17 (68.0)	41 (66.1)	47 (22.0)	**< 0.001**
# of all‐cause death, *n* (%)	34 (11.3)	2 (8.0)	5 (8.1)	27 (12.6)	0.626

^a^ Abbreviations: ADT: androgen deprivation therapy; IQR: interquartile range; N/A: not available; PSA: prostate‐specific antigen.

^b^ PYNEH: sub‐cohort from Pamela Youde Nethersole Eastern Hospital.

^c^ QMH: sub‐cohort from Queen Mary Hospital.

^d^ RJH: sub‐cohort from Ruijin Hospital.

^e^ A two‐tailed *p* value < 0.05 was considered statistically significant; *p* for trend is calculated through the Jonckheere–Terpstra trend test.

**FIGURE 1:**
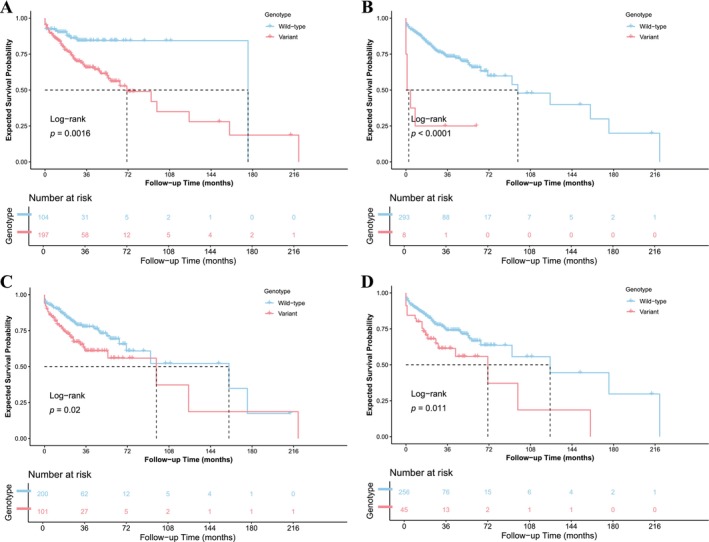
Kaplan–Meier survival curves for significant SNPs under the dominant model. Survival curves illustrating ADT failure‐free survival stratified by genotype groups under the dominant model for (A) rs36119043, (B) rs151155810, (C) rs71179009, and (D) rs28609134.

Multivariate Cox regression analyses were performed in batch for all qualified SNPs, adjusting for Gleason grade group. Clumping was conducted to account for LD, yielding 62 independent clumps. Four intron variants were identified as significantly associated with shortened time to ADT failure (Table 2). These include rs36119043 in *AKR1D1* (hazard ratio, HR = 2.02, 95% confidence interval, 95% CI: 1.44–2.85, *p* = 5.72 × 10^−5^), rs151155810 in *HSD17B12* (HR = 7.87, 95% CI: 2.78–22.30, *p* = 1.05 × 10^−4^), rs71179009 in *SULT2B1* (HR = 2.16, 95% CI: 1.44–3.22, *p* = 1.85 × 10^−4^), rs28609134 in *SRD5A3* (HR = 2.50, 95% CI: 1.51–4.15, *p* = 3.79 × 10^−4^). Kaplan–Meier survival curves were constructed under both dominant (Figure 1) and additive (Figure S2) genetic models of these SNPs.

**TABLE 2:** Results of multivariate Cox regression analyses for SNPs of interest^a^.

SNP	Chromosome	Base pair	Risk allele	Reference allele	HR [95% CI]^b^	*p* ^c^	eQTL gene^d^	NES^e^ [95% CI]	eQTL *p*	eQTL *p* threshold	eQTL tissue
rs36119043	7	137,772,060	—	CT	2.02 [1.44, 2.85]	**5.72 × 10** ^**−5**^	*AKR1D1*	No significant eQTLs.			
rs151155810	11	43,720,433	T	C	7.87 [2.78, 22.30]	**1.05 × 10** ^**−4**^	*HSD17B12*	N/A			
rs71179009	19	49,064,723	—	T	2.16 [1.44, 3.22]	**1.85 × 10** ^**−4**^	*SULT2B1*				
rs28609134	4	56,234,317	C	G	2.50 [1.51, 4.15]	**3.79 × 10** ^**−4**^	*SRD5A3*	−0.26 [−0.36, −0.17]	**5.23 × 10** ^**−8**^	2.02 × 10^−4^	Fibroblasts
								−0.20 [−0.30, −0.10]	**6.28 × 10** ^**−5**^	1.83 × 10^−4^	Thyroid

^a^ SNP: single nucleotide polymorphism.

^b^ HR: hazard ratio; 95% CI: 95% confidence interval.

^c^ A two‐tailed *p* value < 8.06 × 10^−4^ was considered statistically significant.

^d^ eQTL: expression quantitative trait loci. All eQTL‐related data were derived from the Genotype‐Tissue Expression (GTEx) Portal (https://www.gtexportal.org).

^e^ NES: Normalized effect size.

Considering other relevant covariates, including age at enrollment and baseline PSA levels prior to ADT initiation, Spearman's correlation coefficients were calculated (Table S2). Among these, age at enrollment did not show any correlation with the Gleason grade group. Therefore, a sensitivity analysis adjusting for both Gleason grade group and age at enrollment was performed, yielding consistent results (Table S3).

**FIGURE 2:**
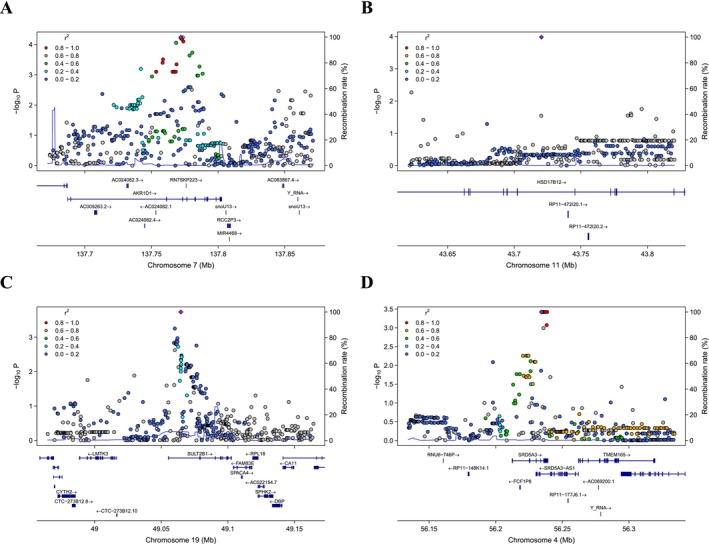
Regional association plots of significant SNPs. Regional plots for the four SNPs significantly associated with time to ADT failure: (A) rs36119043, (B) rs151155810, (C) rs71179009, and (D) rs28609134. The genomic positions are based on Genome Reference Consortium Human Build 37 (GRCh37).

Among these SNPs, rs28609134 was documented in the GTEx database as an eQTL, being significantly associated with the downregulation of *SRD5A3* in fibroblasts and thyroid (Table 2 and Figure S3). We then identified the causal SNPs in the LD regions with r^2^ > 0.8 or D′ > 0.6 within 100 kb of these SNPs of interest (Figure 2). In the LD region of rs36119043, a single nucleotide variant (SNV) rs3735023 located in the 3′‐untranslated region (3′‐UTR) of *AKR1D1* was identified as a potential causal SNP, which is significantly associated with the downregulation of the gene in the adrenal gland. Another synonymous SNV located in the exon of *SULT2B1* has also been discovered to be an eQTL for upregulating the expression of this gene in the LD region of rs71179009 (Table S4). For rs151155810 and rs28609134, most of the SNPs in their LD regions were eQTLs located in the intergenic region in various tissues.

**FIGURE 3:**
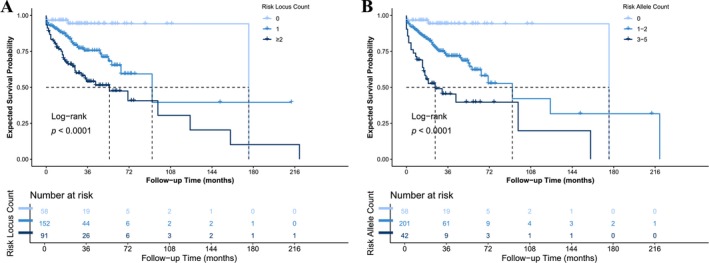
Combined effect of risk alleles on ADT failure. Survival curves comparing ADT failure‐free survival by cumulative number of risk loci or alleles across the four significant SNPs. (A) Under the dominant model. (B) Under the additive model.

A cumulative effect of the four SNPs of interest was also observed, whereby the risk of ADT failure increased with the number of risk loci/alleles carried (Figure 3). Briefly, patients would have 2.74‐fold increased risk (95% CI: 1.86–4.03) of disease progression by each additional risk locus in terms of ADT failure, with a median survival of 176 months (95% CI: N/A) in noncarrier patients vs. 92 months (95% CI: 65‐N/A) in one risk locus‐carriers and 55 months (95% CI: 26‐N/A) in two risk loci‐carriers. Additionally, in the additive model, each additional risk allele would provide a 2.04‐fold (95% CI: 1.63–2.55) increased risk of ADT failure.

## 5 Discussion

In this multicentric cohort study involving Chinese PCa patients receiving ADT, we newly identified four germline SNPs: rs36119043 in *AKR1D1*, rs151155810 in *HSD17B12*, rs71179009 in *SULT2B1*, and rs28609134 in *SRD5A3* that were significantly associated with increased risk of ADT failure. eQTL analyses further revealed that rs28609134, as well as putative causal SNPs in LD with rs36119043 and rs71179009, were probably associated with dysregulation of *SRD5A3*, *AKR1D1*, and *SULT2B1* expression, suggesting the functional relevance of these risk loci in androgen metabolism and PCa treatment response. A cumulative dose–response effect was observed between the number of risk loci/alleles and the hazard of ADT failure, indicating more than a 120‐month difference in median progression‐free survival.

*AKR1D1* is the sole Δ^4^‐3‐ketosteroid‐5β‐reductase in humans, catalyzing the 5β‐reduction of Δ^4^‐3‐ketosteroids, a critical step in bile acid biosynthesis and steroid hormone metabolism [15]. Previous studies have only reported that disease‐causing mutations in *AKR1D1* may lead to bile acid deficiency, whereas its superfamily member *AKR1C* has been implicated in PCa [16]. Our identification of rs36119043 and the functional 3′‐UTR variant rs3735023 in its LD region provides novel evidence implicating the downregulation of *AKR1D1* in adrenal glands during the process of PCa progression under ADT, akin to its *AKR1C* family members.

*HSD17B12* encodes an enzyme involved in processes including the synthesis of arachidonic acid [17]. Previous research has demonstrated that *HSD17B12* upregulation correlates with shorter progression‐free survival in PCa [18]. Multiple *HSD17B12* variants have been discovered to be significantly associated with aggressiveness at diagnosis [19], increased risks of biochemical recurrence, disease progression, and poor overall survival in PCa [20]. Our finding of rs151155810, an intronic SNP associated with adverse ADT response, further underscores its potential as a prognostic biomarker. While relevant eQTLs in its LD region were mostly located in intergenic regions, the underlying regulation deserves future research.

The isoform of the *SULT2B1* gene, *SULT2B1b* catalyzes the sulfation of hydroxysteroids including cholesterol, pregnenolone, and dehydroepiandrosterone (DHEA) [21]. Cholesterol serves as a precursor for steroid hormones, including androgens. *SULT2B1b* is reported to be expressed in reproductive tissues, including the prostate [22]. Previous studies have reported associations of several intronic variants of *SULT2B1* with PCa risk, progression, and overall survival [23, 24]. The identification of rs71179009 in the current study, along with a synonymous exonic variant functioning as an eQTL that increases *SULT2B1* expression, highlights the potential contribution of altered exogenous androgen metabolism to ADT resistance.

*SRD5A3* encodes a type 3 5α‐steroid reductase that catalyzes the conversion of testosterone to dihydrotestosterone (DHT) [25]. Overexpression of *SRD5A3* is characteristic in CRPC cells and metastatic PCa tissues, where its suppression markedly reduces DHT synthesis and cell viability [25, 26]. Previous reports have indicated an early upregulation of *SRD5A3* in PCa patients undergoing ADT, implicating its role in early adaptive resistance mechanisms preceding detectable PSA elevations [27]. Despite our discovery of rs28609134 as an eQTL downregulating *SRD5A3*, this function in tissues apart from the tumor site also suggests a potential impact on systemic androgen metabolism, which may contribute to an altered hormonal environment and influence disease progression beyond the primary tumor microenvironment.

These findings have important clinical implications. The identified germline variants may serve as predictive biomarkers to stratify PCa patients based on their likelihood of responding to ADT, enabling timely adjustment or intensification of hormone therapy based on anticipated disease progression. Nonetheless, functional validation of the identified SNPs was beyond the scope of this study, and replication in independent external cohorts is necessary to confirm their predictive value.

Although the sample size is relatively small and the number of carriers for certain SNPs of interest (e.g., rs151155810) was limited, power analysis indicated that under the Bonferroni‐adjusted type I error level of 8.06 × 10^−4^ (0.05/62 significant SNPs), with the allele frequency in the event being 3.7% and the HR of 7.87, the current cohort may provide sufficient statistical power (100%). In addition, the median follow‐up period of 28 months may limit the detection of late ADT failures among patients with nonmetastatic HSPC, who tend to exhibit longer durations of disease control [28]. This may lead to an underestimation of long‐term treatment failure events. Continued follow‐up of this cohort is ongoing to address this limitation and improve the robustness of future survival analyses. To minimize the confounding effects of other treatment modalities on ADT efficacy, and to exclude cases where ADT was not administered per se for HSPC, we excluded patients who received adjuvant or combination therapies. This highlights the need for future analyses stratified by disease stage and treatment strategy.

Based on these limitations, future research should also focus on validating these findings in larger cohorts with longer follow‐up periods, exploring gene‐internal environment interactions, including immune responses [29], and integrating multi‐omic approaches such as single‐cell omics and spatial omics [30] to better understand the downstream biological pathways involved in explaining both inter‐patient and intra‐tumoral heterogeneity in PCa. Ultimately, these efforts may contribute to the development of precision medicine strategies for PCa management in the Chinese population.

## 6 Conclusion

In this multicentric cohort study, four novel germline SNPs were identified to significantly increase the risk of ADT failure in Chinese PCa patients: rs36119043 in *AKR1D1*, rs151155810 in *HSD17B12*, rs71179009 in *SULT2B1*, and rs28609134 in *SRD5A3*. These findings support their potential as biomarkers for predicting ADT outcomes and guiding personalized treatment strategies.

## Author Contributions

**Ruofan Shi:** data curation, investigation, formal analysis, writing – original draft. **Xiaohao Ruan:** data curation, investigation, resources. **Qijun Du:** data curation, resources. **Tsun Tsun Stacia Chun:** project administration. **Da Huang:** resources, funding acquisition. **Kuen Chan:** resources, data curation. **Yuguang Philip Wu:** resources. **Tsz Yeung Kam:** resources. **Danfeng Xu:** conceptualization, supervision, writing – review and editing. **Rong Na:** conceptualization, supervision, writing – review and editing, funding acquisition.

## Ethics Statement

This study was conducted in accordance with the Declaration of Helsinki, and the study protocol was approved by the Ethics Committee of Shanghai Ruijin Hospital (IRB No. 2020–402, 2 March 2021, version 2), Institutional Review Board of the University of Hong Kong/Hospital Authority Hong Kong West Cluster (IRB No. UW 23–320), and Central Institutional Review Board of Hospital Authority (IRB No. CIRB‐2023‐122‐4). All subjects involved in the study provided informed consent prior to their participation.

## Conflicts of Interest

The authors declare no conflicts of interest.

## Supporting information


**Figure S1.** Flowchart of patient recruitment and selection for the final study cohort. PCa patients who received ADT for HSPC and had available clinical follow‐up and germline SNP genotyping data were identified from three tertiary medical centers. Patients receiving ADT as adjuvant therapy following RP, or in combination with radiotherapy or chemotherapy, were excluded.

**Figure S2.** Kaplan–Meier survival curves for significant SNPs under the additive model. Survival curves illustrating ADT failure‐free survival stratified by genotype groups under the additive model for (A) rs36119043, (B) rs151155810, (C) rs71179009, and (D) rs28609134.

**Figure S3.** eQTL analysis of gene expression stratified by the genotype of rs28609134. Violin plots show normalized expression levels of *SRD5A3* across the different genotypes of rs28609134 in (A) fibroblasts, and (B) thyroid. Data source: GTEx Portal (https://gtexportal.org, Release V10).

**Table S1.** Genes involved in the steroid hormone biosynthesis pathway.

**Table S2.** Spearman correlation between different covariates.

**Table S3.** Sensitivity analysis: multivariate Cox regression adjusted for Gleason grade group and age at enrolment.

**Table S4.** Putative causal eQTL variants in LD regions of SNPs of interest.

## Data Availability

The data that support the findings of this study are available from the corresponding author upon reasonable request.
