# Supplementary material for: Association of Germline Single Nucleotide Polymorphisms in Steroid Hormone Metabolism Pathway With Androgen Deprivation Therapy Prognosis of Prostate Cancer in Chinese Population
Source: Cancer Med. 2025 Nov 2;14(21):e71351. doi: 10.1002/cam4.71351 (PMC12579894; doi:10.1002/cam4.71351)
Supplement: Supplementary file 1 — Figure S1. Flowchart of patient recruitment and selection for the final study cohort. PCa patients who received ADT for HSPC and had available clinical follow‐up and germline SNP genotyping data were identified from three tertiary medical centers. Patients receiving ADT as adjuvant therapy following RP, or in combination with radiotherapy or chemotherapy, were excluded. [file CAM4-14-e71351-s004.pdf]

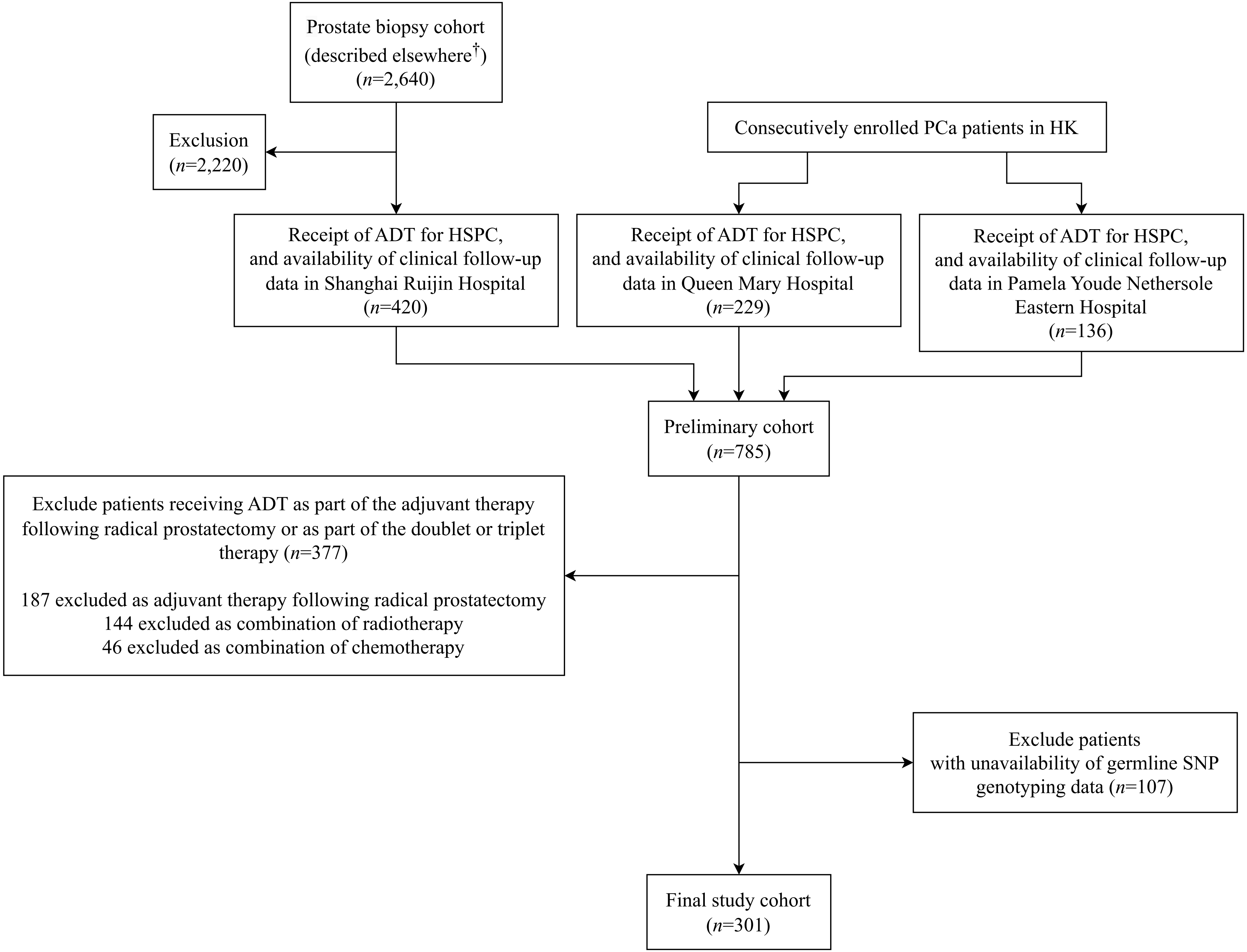

† Wu Y, Ruan X, Gao P, Da H, Fang Z, Xu D, et al. Systematic evaluation of narrow-sense validity of polygenic risk score for prostate cancer in a Chinese prostate biopsy cohort. Clin Genet. 2023;103(6):636-43.
