# Supplementary figures and images for: Association of Germline Single Nucleotide Polymorphisms in Steroid Hormone Metabolism Pathway With Androgen Deprivation Therapy Prognosis of Prostate Cancer in Chinese Population
Source: Cancer Med. 2025 Nov 2;14(21):e71351. doi: 10.1002/cam4.71351 (PMC12579894; doi:10.1002/cam4.71351)

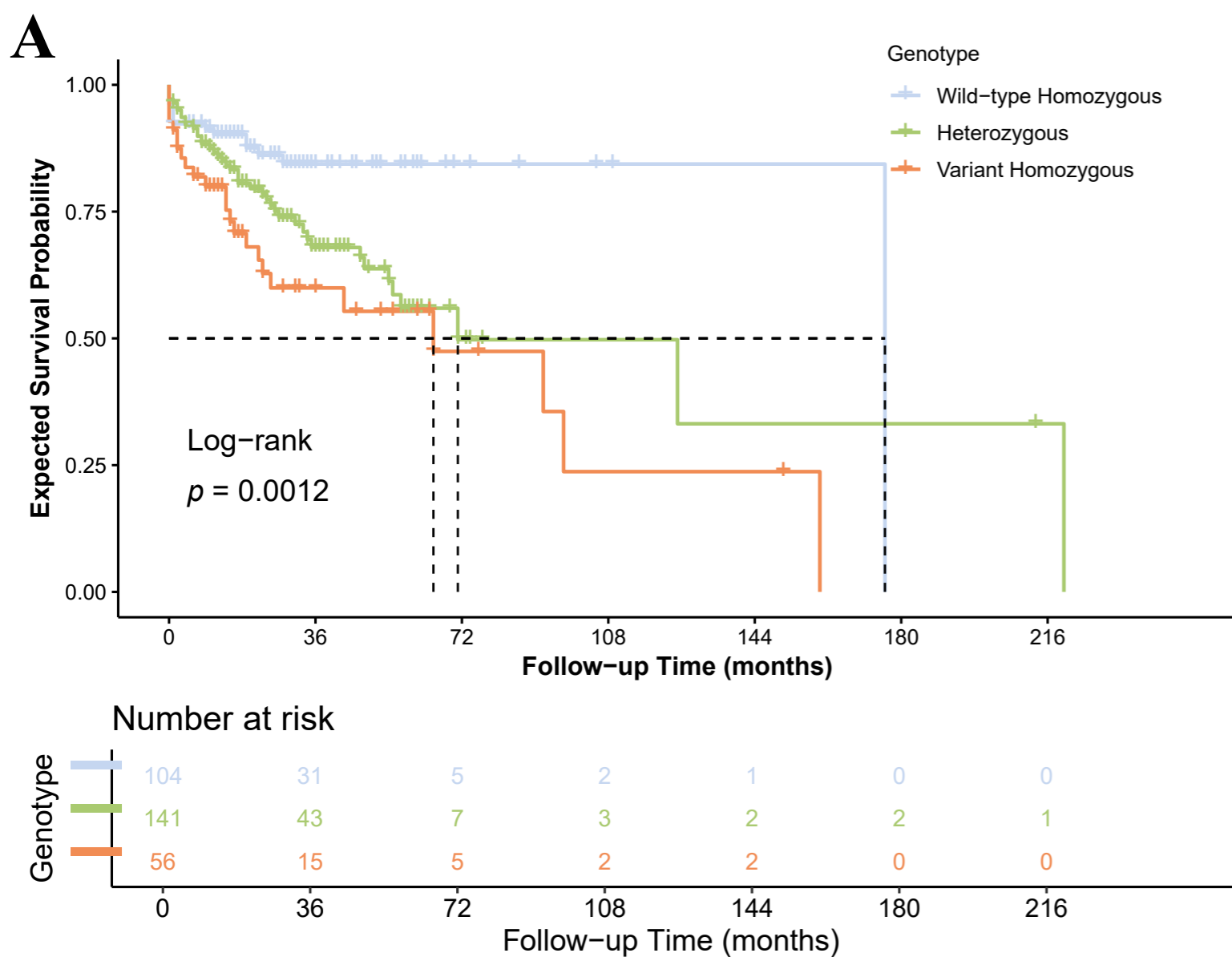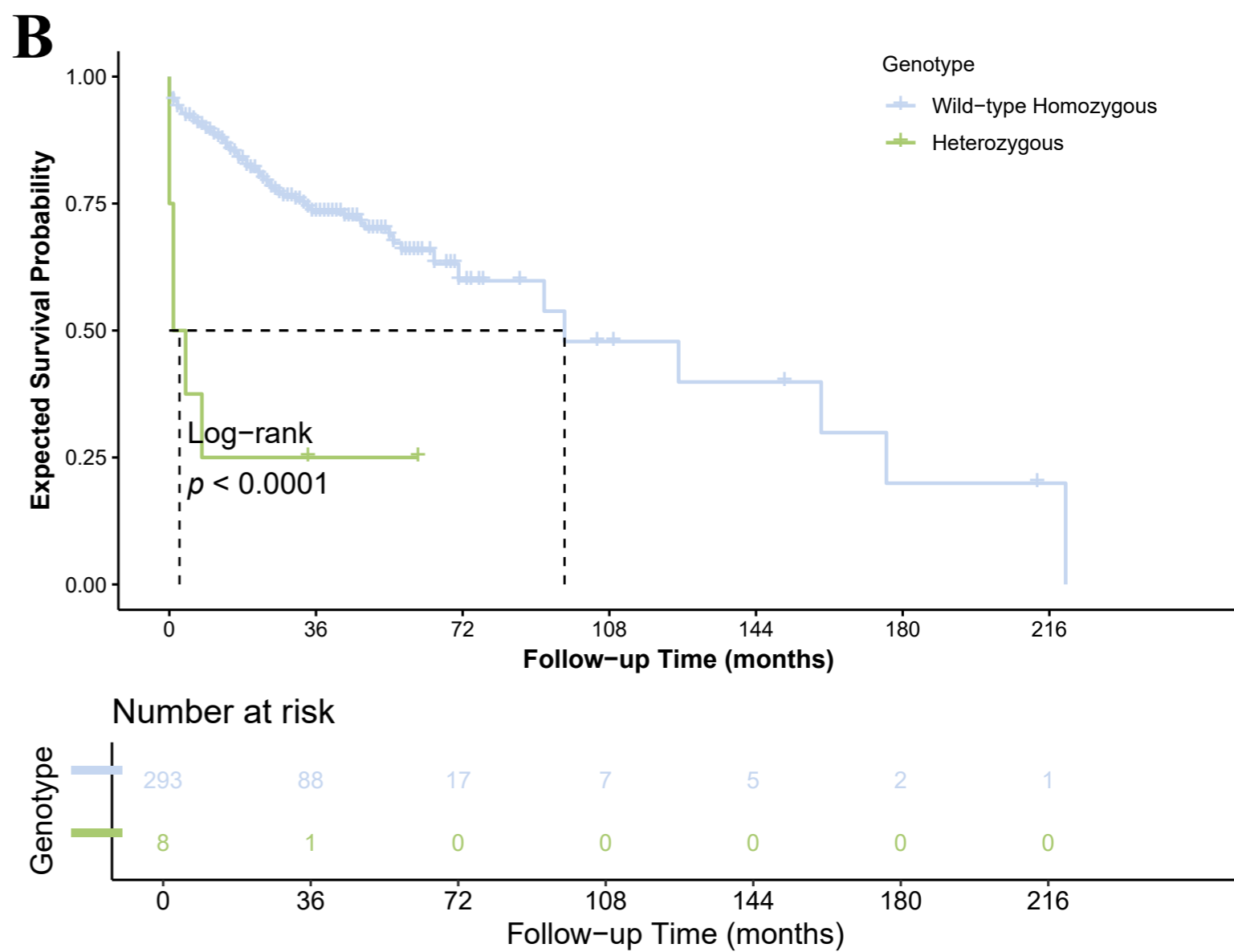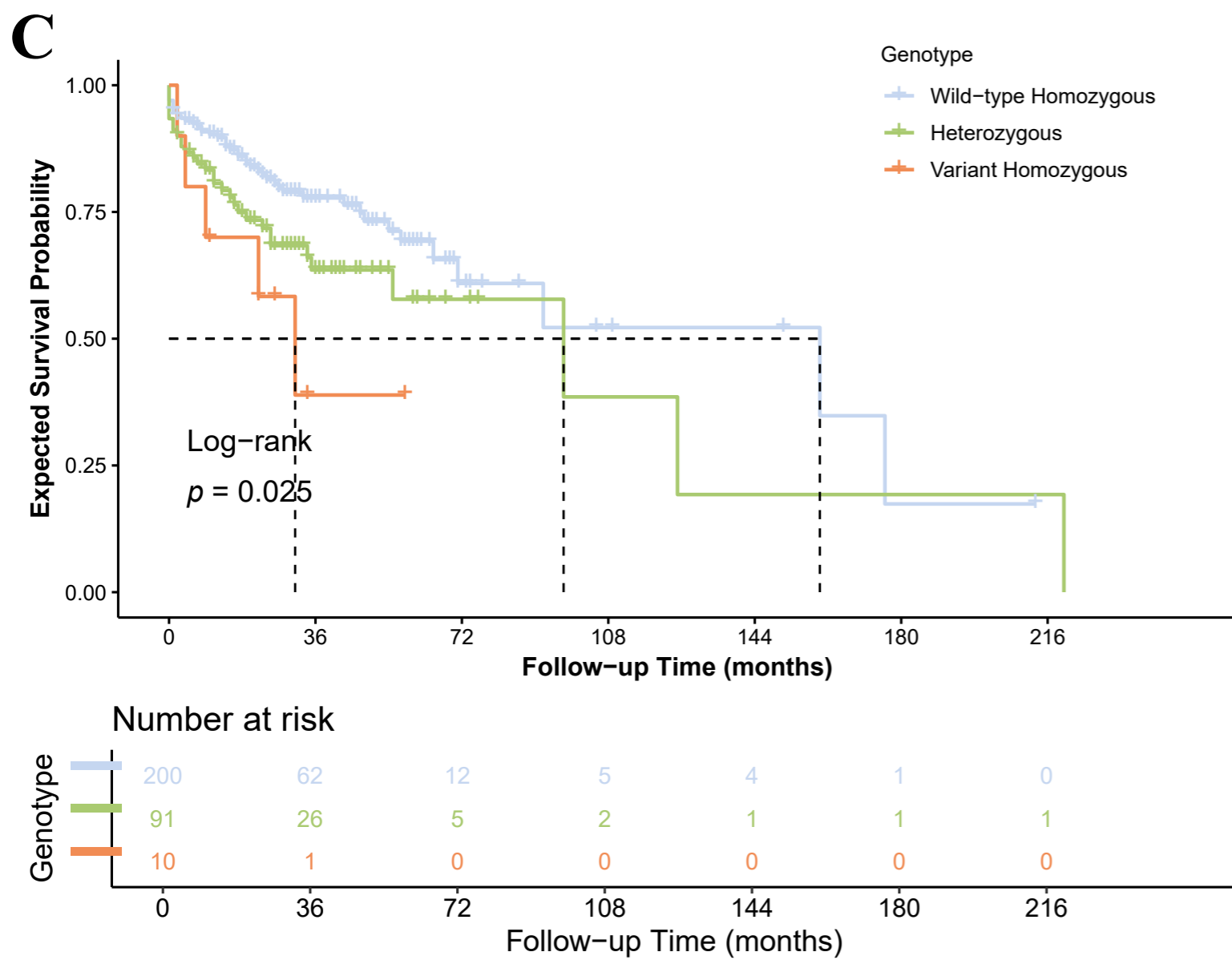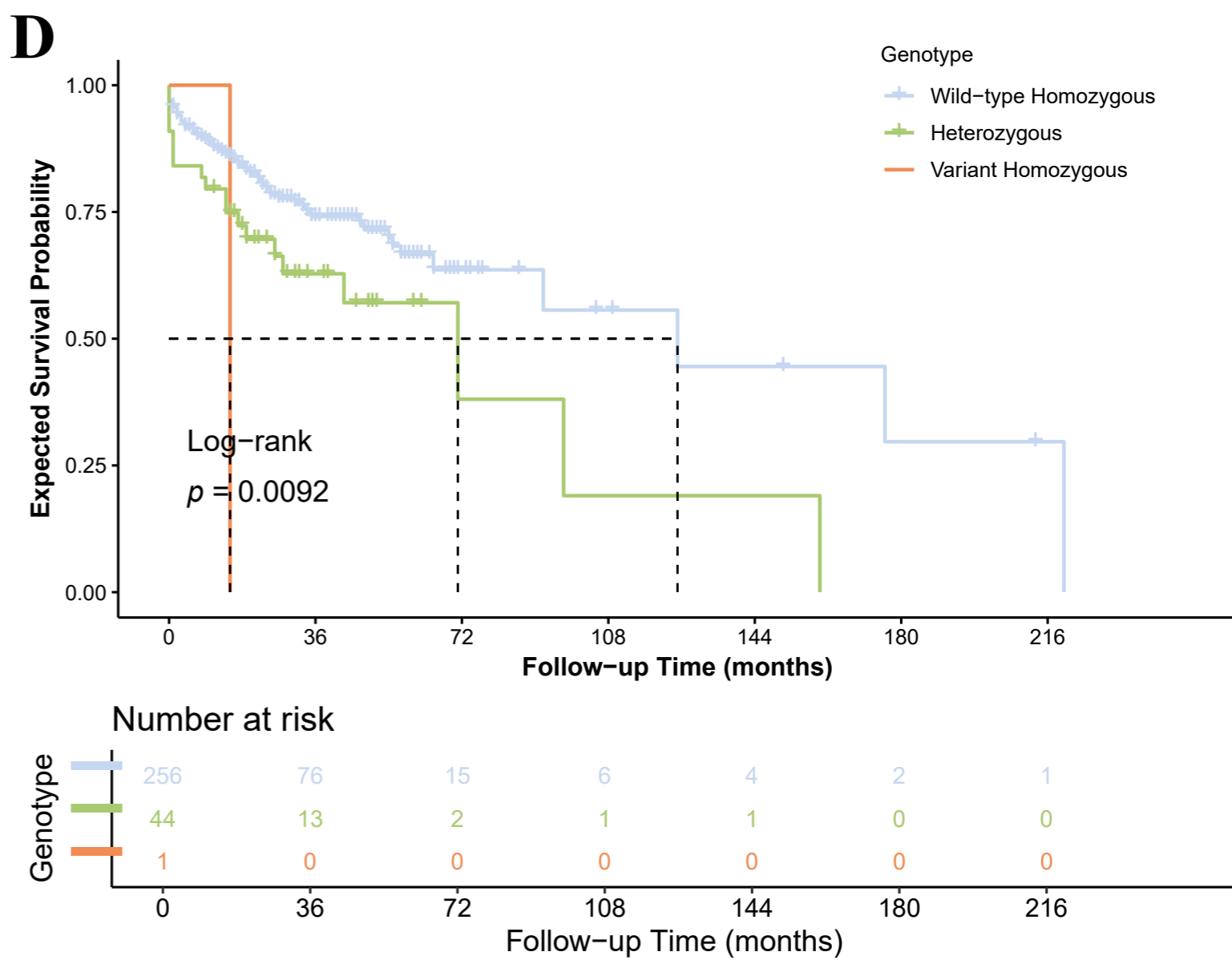

Supplement: Supplementary file 2 — Figure S2. Kaplan–Meier survival curves for significant SNPs under the additive model. Survival curves illustrating ADT failure‐free survival stratified by genotype groups under the additive model for (A) rs36119043, (B) rs151155810, (C) rs71179009, and (D) rs28609134. [file CAM4-14-e71351-s005.pdf]

**A**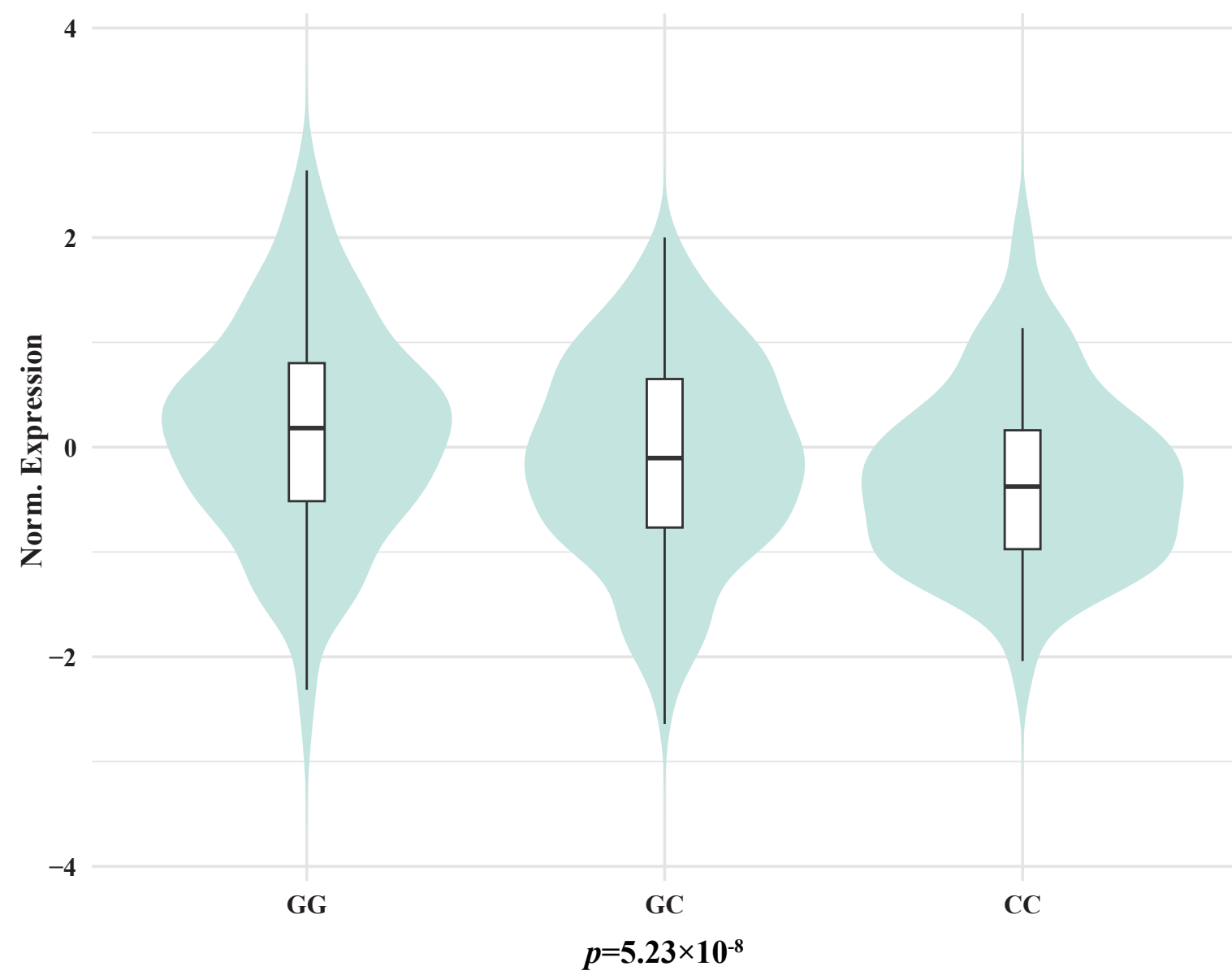**B**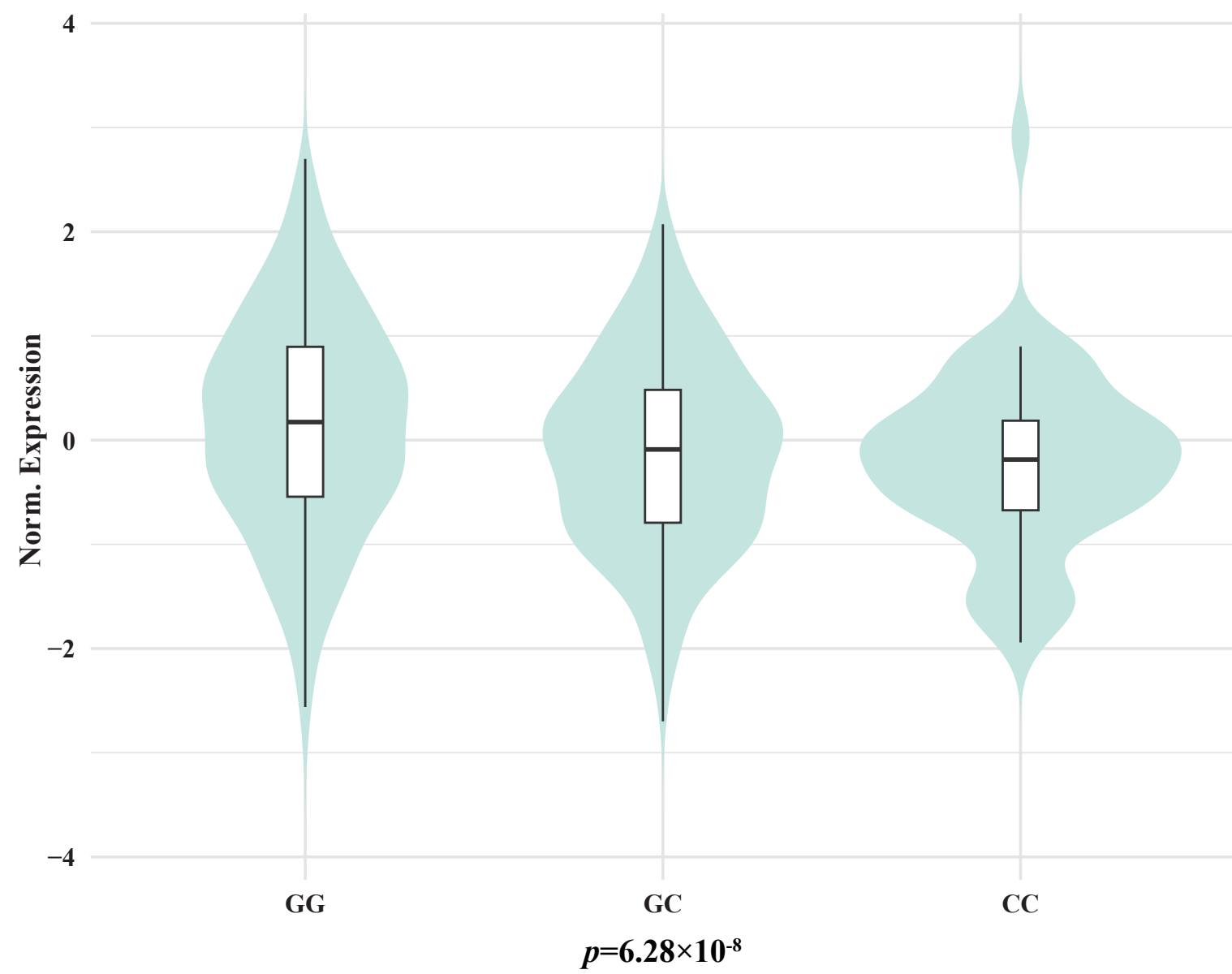

Supplement: Supplementary file 3 — Figure S3. eQTL analysis of gene expression stratified by the genotype of rs28609134. Violin plots show normalized expression levels of SRD5A3 across the different genotypes of rs28609134 in (A) fibroblasts, and (B) thyroid. Data source: GTEx Portal (https://gtexportal.org, Release V10). [file CAM4-14-e71351-s007.pdf]
