# Supplementary material for: Association of Germline Single Nucleotide Polymorphisms in Steroid Hormone Metabolism Pathway With Androgen Deprivation Therapy Prognosis of Prostate Cancer in Chinese Population
Source: Cancer Med. 2025 Nov 2;14(21):e71351. doi: 10.1002/cam4.71351 (PMC12579894; doi:10.1002/cam4.71351)
Supplement: Supplementary file 4 — Table S1. Genes involved in the steroid hormone biosynthesis pathway. [file CAM4-14-e71351-s003.docx]

| **Supplementary Table 1. Genes involved in the steroid hormone biosynthesis pathway** | | | |
| --- | --- | --- | --- |
| **Chromosome** | **Gene Start (hg19)** | **Gene End (hg19)** | **Gene Symbol** |
| 1 | 119957742 | 119989122 | *HSD3B2* |
| 1 | 120049825 | 120057681 | *HSD3B1* |
| 1 | 162760495 | 162782608 | *HSD17B7* |
| 1 | 209859524 | 209908295 | *HSD11B1* |
| 2 | 31749655 | 31806040 | *SRD5A2* |
| 2 | 38294745 | 38303323 | *CYP1B1* |
| 2 | 48913912 | 48982880 | *LHCGR* |
| 2 | 234526290 | 234681951 | *UGT1A8* |
| 2 | 234545122 | 234680261 | *UGT1A1* |
| 2 | 234545122 | 234681951 | *UGT1A10* |
| 2 | 234580543 | 234681951 | *UGT1A9* |
| 2 | 234590583 | 234681951 | *UGT1A7* |
| 2 | 234600320 | 234681951 | *UGT1A6* |
| 2 | 234621637 | 234681951 | *UGT1A5* |
| 2 | 234627437 | 234681951 | *UGT1A4* |
| 2 | 234637772 | 234681951 | *UGT1A3* |
| 4 | 56212387 | 56239266 | *SRD5A3* |
| 4 | 69402902 | 69434245 | *UGT2B17* |
| 4 | 69512314 | 69536494 | *UGT2B15* |
| 4 | 69681712 | 69697741 | *UGT2B10* |
| 4 | 69794176 | 69817509 | *UGT2A3* |
| 4 | 69962192 | 69978705 | *UGT2B7* |
| 4 | 70066050 | 70080449 | *UGT2B11* |
| 4 | 70146216 | 70160768 | *UGT2B28* |
| 4 | 70345882 | 70391732 | *UGT2B4* |
| 4 | 70454134 | 70518967 | *UGT2A1* |
| 4 | 70454134 | 70505360 | *UGT2A2* |
| 4 | 70706929 | 70725870 | *SULT1E1* |
| 5 | 6633499 | 6669675 | *SRD5A1* |
| 6 | 31973358 | 31976712 | *CYP21A1P* |
| 6 | 32006092 | 32009447 | *CYP21A2* |
| 6 | 33172413 | 33174608 | *HSD17B8* |
| 7 | 99245812 | 99332819 | *CYP3A5* |
| 7 | 99302659 | 99332819 | *CYP3A7* |
| 7 | 99354582 | 99381811 | *CYP3A4* |
| 7 | 99425635 | 99464173 | *CYP3A43* |
| 7 | 137687069 | 137790175 | *AKR1D1* |
| 8 | 59402736 | 59412720 | *CYP7A1* |
| 8 | 65508528 | 65711348 | *CYP7B1* |
| 8 | 143953772 | 143961236 | *CYP11B1* |
| 8 | 143991974 | 143999259 | *CYP11B2* |
| 9 | 98997588 | 99064434 | *HSD17B3* |
| 10 | 4934795 | 5020158 | *AKR1C1* |
| 10 | 5005453 | 5149878 | *AKR1C3* |
| 10 | 5029967 | 5060225 | *AKR1C2* |
| 10 | 5238797 | 5260910 | *AKR1C4* |
| 10 | 104590287 | 104597290 | *CYP17A1* |
| 11 | 43702142 | 43878169 | *HSD17B12* |
| 11 | 74811539 | 74875145 | *SLCO2B1* |
| 12 | 20963637 | 21069843 | *SLCO1B3* |
| 12 | 57157107 | 57181574 | *HSD17B6* |
| 12 | 58156116 | 58160976 | *CYP27B1* |
| 15 | 51500253 | 51630795 | *CYP19A1* |
| 15 | 74630102 | 74660081 | *CYP11A1* |
| 15 | 75011882 | 75017877 | *CYP1A1* |
| 16 | 67465035 | 67471454 | *HSD11B2* |
| 16 | 82068857 | 82132139 | *HSD17B2* |
| 17 | 40703983 | 40707232 | *HSD17B1* |
| 19 | 48373722 | 48389654 | *SULT2A1* |
| 19 | 49055428 | 49102684 | *SULT2B1* |
| 20 | 52769987 | 52790516 | *CYP24A1* |
| X | 53458205 | 53461323 | *HSD17B10* |
