# Supplementary material for: Association of Germline Single Nucleotide Polymorphisms in Steroid Hormone Metabolism Pathway With Androgen Deprivation Therapy Prognosis of Prostate Cancer in Chinese Population
Source: Cancer Med. 2025 Nov 2;14(21):e71351. doi: 10.1002/cam4.71351 (PMC12579894; doi:10.1002/cam4.71351)
Supplement: Supplementary file 5 — Table S2. Spearman correlation between different covariates. [file CAM4-14-e71351-s002.docx]

| **Supplementary Table 2. Spearman correlation between different covariates** | | | |
| --- | --- | --- | --- |
| **Covariate A** | **Covariate B^a^** | **Spearman’s *ρ*** | ***p*^b^** |
| Gleason grade group | Age at enrolment | 0.001 | 0.985 |
| Gleason grade group | Serum total PSA before ADT | 0.411 | **<0.001** |
| ^a^ PSA: prostate specific antigen; ADT: androgen deprivation therapy. | | | |
| ^b^ A two-tailed *p* value < 0.05 was considered statistically significant. | | | |
