# Supplementary material for: Association of Germline Single Nucleotide Polymorphisms in Steroid Hormone Metabolism Pathway With Androgen Deprivation Therapy Prognosis of Prostate Cancer in Chinese Population
Source: Cancer Med. 2025 Nov 2;14(21):e71351. doi: 10.1002/cam4.71351 (PMC12579894; doi:10.1002/cam4.71351)
Supplement: Supplementary file 6 — Table S3. Sensitivity analysis: multivariate Cox regression adjusted for Gleason grade group and age at enrolment. [file CAM4-14-e71351-s006.docx]

| **Supplementary Table 3. Sensitivity analysis: multivariate Cox regression adjusted for Gleason grade group and age at enrolment** | | | | | | |
| --- | --- | --- | --- | --- | --- | --- |
| **SNP** | **Chromosome** | **Base Pair** | **Risk Allele** | **Reference Allele** | **HR [95% CI]^a^** | ***p*^b^** |
| rs36119043 | 7 | 137772060 | - | CT | 2.03 [1.44, 2.87] | **5.31×10^-5^** |
| rs151155810 | 11 | 43720433 | T | C | 7.84 [2.75, 22.30] | **1.15×10^-4^** |
| rs71179009 | 19 | 49064723 | - | T | 2.15 [1.44, 3.21] | **1.98×10^-4^** |
| rs28609134 | 4 | 56234317 | C | G | 2.51 [1.51, 4.19] | **4.02×10^-4^** |
| ^a^ HR: hazard ratio; 95% CI: 95% confidence interval. | | | | | | |
| ^b^ A two-tailed *p* value < 6.76×10^-4^ was considered statistically significant. | | | | | | |
