# Supplementary material for: Association of Germline Single Nucleotide Polymorphisms in Steroid Hormone Metabolism Pathway With Androgen Deprivation Therapy Prognosis of Prostate Cancer in Chinese Population
Source: Cancer Med. 2025 Nov 2;14(21):e71351. doi: 10.1002/cam4.71351 (PMC12579894; doi:10.1002/cam4.71351)
Supplement: Supplementary file 7 — Table S4. Putative causal eQTL variants in LD regions of SNPs of interest. [file CAM4-14-e71351-s001.docx]

| **Supplementary Table 4. Putative causal eQTL variants in LD regions of SNPs of interest^a^** | | | | | | | | | | |
| --- | --- | --- | --- | --- | --- | --- | --- | --- | --- | --- |
| **SNP** | **Chromosome** | **Base Pair** | **Alternate Allele** | **Reference Allele** | **Function** | **eQTL Gene** | **NES^b^ [95% CI]** | **eQTL *p*** | **eQTL *p* Threshold** | **eQTL Tissue** |
| rs3735023 | 7 | 137801770 | C | A | 3’-UTR variant | *AKR1D1* | -0.56 [-0.74, -0.40] | **7.56×10^-10^** | 3.60×10^-5^ | Adrenal gland |
| rs2544794 | 19 | 49079246 | A, G, T | C | Synonymous variant | *SULT2B1* | 0.37 [0.25, 0.48] | **9.54×10^-10^** | 5.26×10^-5^ | Stomach |
| ^a^ eQTL: expression quantitative trait locus; SNP: single nucleotide polymorphism. All eQTL-related data were derived from the Genotype-Tissue Expression (GTEx) Portal (https://www.gtexportal.org). | | | | | | | | | | |
| ^b^ NES: Normalized effect size. | | | | | | | | | | |
